# Study the Features of 57 Confirmed CRISPR Loci in 38 Strains of *Staphylococcus aureus*

**DOI:** 10.3389/fmicb.2018.01591

**Published:** 2018-07-26

**Authors:** Xihong Zhao, Zhixue Yu, Zhenbo Xu

**Affiliations:** ^1^Research Center for Environmental Ecology and Engineering, Key Laboratory for Green Chemical Process of Ministry of Education, Key Laboratory for Hubei Novel Reactor & Green Chemical Technology, School of Environmental Ecology and Biological Engineering, Wuhan Institute of Technology, Wuhan, China; ^2^School of Food Science and Engineering, South China University of Technology, Guangzhou, China

**Keywords:** CRISPR, direct repeat, *Staphylococcus aureus*, spacer, cas, food safety

## Abstract

*Staphylococcus aureus* is a foodborne pathogen that causes food contamination and food poisoning, which poses great harm to health, agriculture and other hosts. Clustered regularly interspaced short palindromic repeats (CRISPR) are a recently discovered bacterial immune system that resists foreign genes such as phage DNA. This system inhibits the transfer of specific movable genetic elements that match the CRISPR spacer sequences, thereby preventing the spread of drug-resistant genes between pathogens. In this study, 57 CRISPR loci were screened from 38 strains of *S. aureus* based on the CRISPR database, and bioinformatics tools were used to investigate the structural features and potential functions of *S. aureus* CRISPR loci. The results showed that most strains contained only one CRISPR locus, a few strains contained multiple loci with sparsely distributed sites. These loci mainly included highly conserved direct repeat sequences and highly variable spacer sequences, as well as polymorphic *cas* genes. In addition, the analysis of secondary structure of direct repeat RNA showed that all sites can form stable RNA secondary structure. The results of constructing phylogenetic tree based on spacer sequence showed that some strains contained a high degree of phylogenetic relationship, while the differences among other strains in evolutionary processes were quite obvious. Of the 57 CRISPR loci identified, only the *cas* gene was found near the 4 CRISPR loci.

## Introduction

In the past, it was known that vertebrates have the ability to resist and eliminate foreign pathogens, and could form a highly effective secondary immune mechanism to prevent the re-invasion of pathogens. Until the advent of clustered regularly interspaced short palindromic repeats (CRISPR), researchers realized that prokaryotes also had an adaptive secondary immune system similar to animals (Morange, 2015). CRISPR was the product of the evolution of life in the history of bacterial invasion against viruses, the bacteria to remove the virus invasive alien genes, evolved this powerful immune defense system. The main fundamental principle of the CRISPR system: firstly, the characteristic gene (proto-spacer) was extracted from the invaded foreign DNA and embedded in the CRISPR locus. Secondly, when the exogenous phage invaded again, this prokaryotic immune system used characteristic genes (spacers) to rapidly target and recognized foreign DNA. Finally, with the participation of a Cas protein complex, the invading phage DNA sequence was targeted and interfered, and the recognized foreign DNA was excised to eliminate exogenous invasion. The prokaryotic immune system, both acquired and heritable, is widespread in about 47% of bacteria and 84% of archaebacterial, which truly documents the pathways of bacterial evolution and the history of confrontations with foreign invaders (such as phages) (Lillestøl et al., 2006; Wakefield et al., 2015; Yang et al., 2015). *Staphylococcus aureus*, as an important foodborne pathogen in humans, is widely found in nature, air, water, dust, and human and animal excrement (Zhao et al., 2016). Furthermore, *S. aureus* is the most common pathogen in human purulent infection, in addition to local purulent infection, but also can cause pneumonia, pseudomembranous colitis, pericarditis, and even systemic infection such as sepsis (Larkin et al., 2009; Lindsay, 2014; Pérez-Montarelo et al., 2017). The pathogenicity of *S. aureus* mainly depends on the toxins and invasive enzymes it produces (Zhao X. H. et al., 2017). One of the most important toxins is enterotoxin, a protein toxin that can cause acute gastroenteritis. The enterotoxin can tolerate boiling at 100°C for 30 min without being destroyed, and the food poisoning symptoms causes are vomiting and diarrhea (Zhang and Zhao, 2017; Zhang et al., 2017; Zhao Y. et al., 2017). At present, *S. aureus* can be divided into five groups and 26 types using phage typing (Dua et al., 1982), most of which have high environmental adaptability. It is tempting to speculate that CRISPR, as prokaryotic immune system, may be closely related to the high environmental adaptability of *S. aureus* (Schröder et al., 2013).

The typical genomic architecture of a CRISPR-Cas system is consists of a CRISPR locus, a series of *cas* genes, and a leader region. One of the major system components is the CRISPR locus, which is characterized by a series of tandem repeats separated with a unique spacer sequence (Horvath and Barrangou, 2010; Koonin and Makarova, 2013). The spacers of the CRISPR locus are highly specific, while the repeats are almost identical in same CRISPR locus (Guzina et al., 2017; Rossi et al., 2017). The direct repeat contains palindrome, which can form RNA secondary hairpin structure. Further study found that the hairpin structure was the recognition and binding site of Cas protein in the process of interference function (Wang et al., 2011). The spacer sequences were shown to be derived from previously encountered phages (Bolotin et al., 2005), and a small proportion came from the same bacterial genome, suggesting that there is horizontal gene transfer between homologous species in CRISPR system (Grissa et al., 2008; Rossi et al., 2017). In addition to the CRISPR locus, CRISPR-Cas system also includes a series of Cas proteins with multiple nuclease activities, as well as the leader region identified as having promoter function. In general, the CRISPR-Cas system can be divided into two categories based on the Cas protein. Class 1 systems perform functions through a multi-subunit Cas protein complex, whereas the Class 2 systems only require a single Cas protein (Cas9 or Cpfl) in the crRNA effector complex. Class 1 includes Type I, Type III, and Type IV systems, and Class 2 includes Type II and Type V systems (Quan and Ye, 2017). It illustrates the complexity of the CRISPR system from another perspective.

At present, traditional pathogen detection methods include PCR, bacterial identification media, latex agglutination test, and traditional bacterial typing methods such as serotyping, phage typing, and drug resistance profile typing (Sabat et al., 2013; Zhong and Zhao, 2017, 2018; Wei et al., 2018; Zhao et al., 2018). It has been difficult for them to meet the current requirements for accurate diagnosis, traceable typing, and epidemiological studies of pathogens. For instance, foodborne pathogens once induced into the viable but non-culturable state (VBNC) cannot be detected by routine bacterial culture assays and can easily lead to undetected pathogens caused by the VBNC status, posing a serious threat to food safety and human health (Zhao et al., 2014; Ding et al., 2017; Liu et al., 2017; Zhao X. et al., 2017). Therefore, exploring how to identify pathogens more accurately and rapidly at the genetic level becomes a new direction for CRISPR systems in the field of microbiology. Currently, researchers have proposed to apply the diversity and specificity of spacers in CRISPR to genotyping techniques and have been well-established in some bacteria. For instance, Shariat et al. (2013) have proposed a new typing method CRISPR-MVLST which was a combination of bacterial marker gene, CRISPR and multiple site sequence typing. This typing method not only showed high degree consistency in epidemiology, but also higher resolution than pulsed-field gel electrophoresis, which distinguished highly cloned strains during pathogen outbreaks. In fact, the focus of CRISPR research is very prominent, especially as a faster and simpler application of gene editing tools (Doudna and Charpentier, 2014; Zhang et al., 2014), which can be used in biotechnology and medicine, and has great potential in gene and cell therapy (Maeder and Gersbach, 2016). The third generation of gene editing CRISPR-Cas9 technology is used to study the genetic engineering of various organisms such as eukaryotes, bacteria and viruses (Penewit et al., 2018). As CRISPR technology continues to improve, replacing Cas9 with Cpf1 or xCas9 may provide more opportunities for these applications (Hu et al., 2018). Given the diversity of CRISPR-Cas systems in different prokaryotes (Koonin et al., 2017; Wexler and Tajkarimi, 2017), researchers have been working on these sites in various bacteria in recent years. At present, bioinformatics tools can help researchers expand their knowledge in different fields without the need for laborious laboratory experiments. Based on this method, there have been some studies on the CRISPR system in several bacteria (Hidalgo-Cantabrana et al., 2017; Negahdaripour et al., 2017; Tomida et al., 2017; Hao et al., 2018). Bioinformatics Analysis of CRISPR in foodborne pathogens is crucial for assessing the potential evolution of foodborne pathogens to predict the outbreak of food borne pathogens, which is of great importance to food safety. In order to investigate the presence of CRISPR in different strains and the range of possible immune defenses, the effects of different CRISPR/Cas systems on the pathogenicity of *S. aureus* were explored, and accurately and quickly detect foodborne pathogenic bacteria, prevent and eliminate *S. aureus* caused by a variety of foodborne infections. It is significant to decipher the CRISPR locus that has now been identified. Therefore, systematic research on CRISPR structures helps us to better explore other functions of the CRISPR system in addition to bacteriophage immunity.

In this study, 57 identified CRISPR loci from 38 species of *S. aureus* were selected for experimental subjects. By bioinformatics analysis of its structural characteristics and potential functional activity in *S. aureus*, based on the similarity of the phylogenetic relationships between spacer and direct repeat sequences, the 57 CRISPR loci were classified and verified that the spacer sequence was derived from a phage or exogenous plasmid. In addition, bioinformatics tools were used to predict RNA secondary structure formed by direct repeat sequences and their stability. Finally, the presence and distribution of the *cas* gene near the CRISPR locus were analyzed. The aim is to provide a new strategy for the control of foodborne pathogens *S. aureus* resistance studies, genotyping, traceability analysis, food safety and prevention.

## Materials and methods

### Data sources

The different *S. aureus* strain genomes were searched by the National Center for Biotechnology Information (NCBI) nucleotide database (http://www.ncbi.nlm.nih.gov/) with default parameters; then *S. aureus* CRISPR loci were searched by the CRISPR Finder server (*E*-value ≤ 0.001) (http://crispr.i2bc.paris-saclay.fr/Server/)(Last updated on May 9, 2017). The CRISPR loci contained in 38 stains of *S. aureus* were classified as suspected CRISPR loci and identified CRISPR loci. All the identified 57 CRISPR loci were selected for study.

### Analysis method

CRISPR loci were grouped according to the similarity of the common direct sequence repeats (CDRs) of the CRISPR locus. First, using the MEGA6.06 software (https://www.megasoftware.net/) for direct sequences multiple sequence alignment (MSA), similar repeat sequences were clustered into the same group, and then the RNA secondary structures and minimum free energy (MFE) of each direct repeat sequence were predicted by the RNA fold Web server (http://rna.tbi.univie.ac.at/cgi-bin/RNAWebSuite/RNAfold.cgi). As for the algorithm for secondary structure folding and MFE, the output option was set to default. The prediction of these structures are based on the cyclic energy model and the dynamic program definition algorithm (Zuker and Stiegler, 1981).

In addition, since the CRISPR system may contain numerous spacers, the principal component analysis (PCA) was used to screen representative spacers from each CRISPR system, then MEGA6.06 software (https://www.megasoftware.net/) was used to import the selected spacer sequences to describe phylogenetic tree and identify the phylogenetic relationships among the stains, the genotyping and phylogenetic relationship of 38 strains of *S. aureus* were predicted, and the highly homologous sequence sources were searched by NCBI BLASTN search pattern (default parameters, nr database, mismatches ≤ 3). Finally, the *cas* gene near the CRISPR locus was searched in CRISPRs database (http://crispr.i2bc.paris-saclay.fr/crispr/). In addition, multiple sequence aligned proteins were searched by the BLASTP platform in NCBI (identity ≥ 90%, coverage > 90%). The above results are used to describe the type of CRISPR system and the distribution of *cas* genes in order to achieve bioinformatic analysis of the CRISPR locus identified in the selected strain.

## Results

### CRISPR locus of *Staphylococcus aureus* in CRISPR database

CRISPR database is a relational database implemented using mysql 4.1 (http://www.mysql.com/). Its implementation of related Web services is based on Perl 5.8.8 (http://www.perl.org/) and the Linux operating system (debian Sarge 3.1). Run on the Apache 2.0 web server (http://www.apache.org/). And use some modules to process the sequence. The database core application consists of two main programs: (a) CRISPR Finder for detecting CRISPRs and extracting them from the genome sequence. (b) Database tools for downloading prokaryotic genomes from the NCBI ftp site (ftp://ftp.ncbi.nih.gov/genomes/Bacteria), save CRISPRs, and update them (Grissa et al., 2007a). Currently, CRISPR database analyzes the genomes of 232 archaea by CRISPR Finder and obtains 870 CRISPR loci, 202 of which are convincing. In addition, 8,690 CRISPR loci were obtained by analyzing the genomes of 6,786 bacterial species, of which 3,059 loci were identified. In the CRISPR database, CRISPR loci contained in 38 *S. aureus* were searched. Among them, 57 CRISPR loci have been confirmed and others were suspicious CRISPR loci (last updated: 9 May, 2017), 38 strains of *S. aureus* have been confirmed in 57 CRISPR loci to carry out bioinformatics analysis of the components. As shown in Table 1, 22 strains of *S. aureus* contained only one CRISPR locus, 14 strains of *S. aureus* contained 2 CRISPR loci, and the other 2 strains contained 3 and 4 CRISPR loci. This was in comparison to other CRISPR loci on the distribution of the number of species was rare, it came down to the fact that the study of the *S. aureus* CRISPR system was not extensive enough, there may be some CRISPR loci hidden in the large number of suspicious CRISPR sequences. The number of spacer sequences in each CRISPR loci was between 1 and 15, the length of spacer sequence was concentrated in 25–31 bp, the number of direct repeats was between 2 and 16, and the length of the sequence was concentrated in 23–26 bp.

**Table 1:** Statistical table of 38 CRISPR loci of *Staphylococcus aureus*.

**Stain**	**Genbank ID**	**Source**	**CRISPR ID**	**Number of CRISPR**	**Number of spacers**	**CRISPR length**	**DR length**	**Spacer length**
*S. auras* 04_02981	387149188	Nübel et al., 2010	NC_017340_5,10	2	1, 1	78, 80	23, 25	33, 31
*S. auras* 08BA02176	404477334	Golding et al., 2012	NC_018608_1,2	2	15, 2	1107, 183	36, 38	36, 34, 34 36, 37, 34 37, 35, 38 37, 35, 36 35, 33, 35, 34, 36
*S. auras* GCF_000597965	749295051	Parker et al., 2014	NZ_CP007454_4,5	2	1,1	78, 80	33, 25	33, 31
*S. auras* GCF_000695875	749295046	Sabirova et al., 2014	NZ_CP007690_7	1	1	78	23	33
*S. auras* GCF_000815045	749198600	Daum et al., 2015	NZ_CP010295_6	1	1	78	23	33
*S. auras* GCF_000815085	749203622	Daum et al., 2015	NZ_CP010296_6	1	1	78	23	33
*S. auras* GCF_000815165	749193063	Daum et al., 2015	NZ_CP010298_6	1	1	78	23	33
*S. auras* GCF_000969225	806462661	Mcculloch et al., 2015	NZ_CP011147_4,10	2	1,1	78, 80	23, 25	33, 31
*S. auras* GCF_001021895	829615601	Tenover and Goering, 2009	NZ_CP007674_3,8	2	1,1	80, 78	26, 23	29, 33
*S. auras* GCF_001045795	983310191	Planet et al., 2015	NZ_CP007672_5	1	1	78	23	33
*S. auras* GCF_001278745	983466147	Panesso et al., 2015	NZ_CP012593_6,12	2	1,1	78, 80	23,25	33, 31
*S. auras* GCF_001281145	927544131	Giraud et al., 2015	NZ_CP010890_5,10	2	1,1	78, 80	23, 25	33, 31
*S. auras* GCF_001457495	983361205	Holmes et al., 2016	NZ_LN831036_4	1	1	84	26	33
*S. auras* GCF_001465755	983420869	Bosch et al., 2016	NZ_CP013621_1	1	3	199	31	25, 25, 26
*S. auras* GCF_001558795	1344139377	Giannuzzi et al., 1999	NZ_CP014064_1,4	2	1,1	78,80	23, 25	33, 31
*S. auras* GCF_001594205	1008818213	Aswani et al., 2016	NZ_CP014791_5	1	2	133	23	32, 33
*S. auras* GCF_001611345	1016065235	Trouilletassant et al., 2016	NZ_CP012978_1,3,4,6	4	1,2,4,1	80, 136, 250, 82	26, 24, 27, 26	29, 33, 32, 29, 29, 29, 29, 31
*S. auras* GCF_001611385	1016064704	Trouilletassant et al., 2016	NZ_CP012974_3,4,9	3	1,1,2	78, 81, 136	23, 26, 25	33, 30, 31 31
*S. auras* GCF_001611425	1016068196	Trouilletassant et al., 2016	NZ_CP012970_1,4	2	1,1	80, 82	26, 26	29, 31
*S. auras* GCF_001641025	1027722058	Tatusova et al., 2013	NZ_CP013957_4	1	1	80	26	29
*S. auras* GCF_001725965	1065087359	Lim et al., 2015	NZ_CP012692_5,12	2	1,1	79, 80	24,25	32, 31
*S. auras* GCF_900092595	1045302382	Maël et al., 2016	NZ_LT598688_6	1	1	78	23	33
*S. auras* subs. auras 11819-97	385780298	Stegger et al., 2012	NC_017351_4	1	1	84	26	33
*S. auras* subs. auras 71193	386727822	Uhlemann et al., 2012	NC_017673_1	1	3	199	31	25, 25, 26
*S. auras* subs. auras ACT-R 2	384863396	Lindqvist et al., 2012	NC_017343_3,8	2	1,1	78, 80	23,25	33, 31
*S. auras* subs. auras ED133	384546269	Guinane et al., 2010	NC_017337_9	1	1	78	24	31
*S. auras* subs. auras GCF_000772025	755010342	Lim et al., 2015	NZ_CP009554_7	1	1	80	25	31
*S. auras* subs. Auras GCF_001296985	930161532	Sabat et al., 2015	NZ_CP010402_3	1	1	84	26	33
*S. auras* subs. auras GCF_001515665	975875548	Botelho et al., 2016b	NZ_CP012015_4	1	1	80	26	29
*S. auras* subs. auras GCF_001515705	975875579	Botelho et al., 2016a	NZ_CP012018_4	1	1	80	26	29
*S. auras* subs. auras GCF_001515765	975883094	Costa et al., 2013	NZ_CP012012_3	1	1	80	26	29
*S. auras* subs. auras HO 5096 0412	386829725	Holden et al., 2013	NC_017763_1	1	2	137	27	28 29
*S. auras* subs. auras JH1	150392480	Kim et al., 2014	NC_009632_4,9	2	1, 1	78, 80	23, 25	33, 31
*S. auras* subs. auras MSHR1132	379794527	Holt et al., 2011	NC_016941_1,2	2	6, 4	469, 311	36, 23	36, 37, 37, 34, 36, 38, 48, 49, 51, 49
*S. auras* subs. auras T0131	384868588	Li et al., 2011	NC_017347_9	1	1	78	23	33
*S. auras* subs. auras USA300	87159884	Diep et al., 2006	NC_007793_6	1	1	78	23	33
*S. auras* subs. auras VC40	379013365	Sass et al., 2012	NC_016912_6	1	1	78	23	33
*S. auras* subs. auras Z172	554642795	Chen et al., 2013	NC_022604_4,7	2	1,2	80, 138	26, 28	29, 29, 28

### Repeat sequences

**Figure 1:**
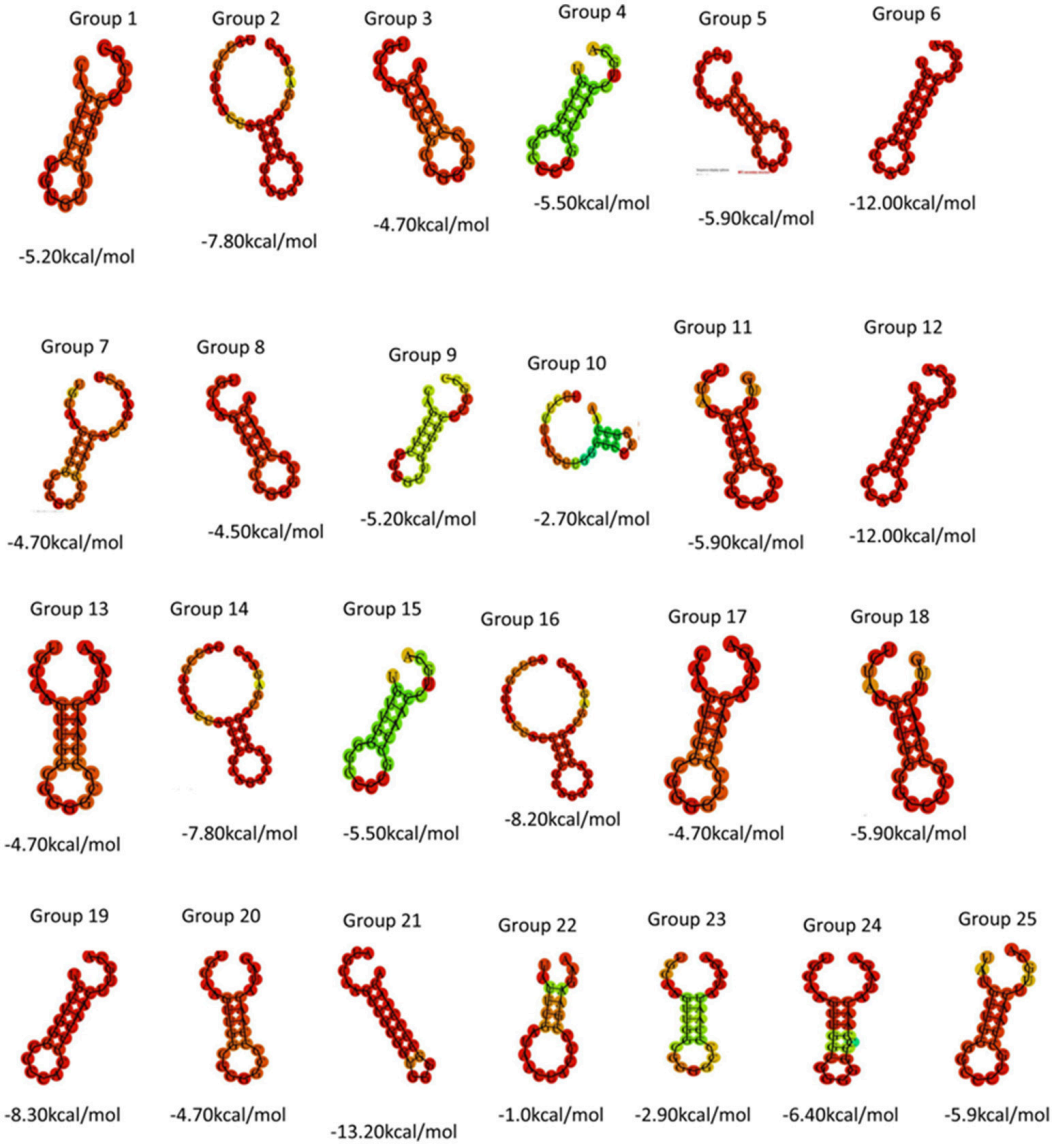
Using DR sequences to generate 25 sets of RNA secondary structure prediction and MFE values.

Direct repeat sequences (DR) are always highly similar or identical at the same CRISPR locus, the consensus direct repeat sequence (CDR) of each CRISPR locus for multiple sequence alignment analysis was chosen. Based on the results of the comparison, 57 CRISPR loci in 38 strains of *S. aureus* were divided into 25 groups. As shown in Table 2, each group was composed of the same DR sequence. In order to facilitate multiple sequence comparison and clustering, one DR sequence for homology analysis was chosen in each group. In addition, the RNA secondary structure of each repeat of 25 sets of DR sequences was predicted and recorded its MFE by RNA fold web server (Grissa et al., 2007b). In all groups, the RNA secondary structure was bound at both ends, forming stems in the middle. As shown in Figure 1, group 2, group 14, group 16, and group 22 were predicted to have a repeat length of 4 bp for the RNA secondary structure, 3 bp for the 9th group, the stem length of the secondary structure of group 19 and group 24 was 6 bp, the length of the 6th and 12th groups was 7 bp, the length of the secondary structure formed by group 21 was 8 bp, and that of other groups was 5 bp. Due to the formation of difference algorithm and system structure uncertainty, in partial red graphic representation form different secondary structure types, the structure formation probability prediction system was the possibility of relatively large, While the greenish-green indicated that the RNA secondary structure formed by the repeated sequences was still predicted to have a low probability of formation after the system optimization algorithm (Mathews, 2004). In addition, the sequence stability and the degree of conservation of the DR can be predicted by RNA secondary structure diagram and MFE value. Due to possible systematic errors in the prediction of the green group with a lower probability of being predicted, the group with the largest MFE value was group 22, which meant that it was not only the most unstable in the green marker group but also the least stable in all RNA secondary structures. The 21th group had a minimum MFE value of −13.2 kcal/mol and the longest stem formed in all RNA secondary structures, which was in line with the secondary structure of RNA stability and the formation of the stem length showed a certain linear positive correlation theory. In the same cluster, in addition to stem length can affect the stability of the secondary structure; other factors include the length of the repeat sequence and the “GC” content. In general, repeats with higher “GC” content at the same length are more stable; the longer the repeats, the more stable the secondary structure may be. Overall, the DR sequence with lower MFE value was more stable than the DR sequence with high MFE value.

**Table 2:** DR Sequence and RNA secondary structure MFE value statistics.

**Group**	**DR Consensus**	**Number of CDR**	**Percentage (%)**	**Min Free Energy (kcal/mol)**
1	CAGCTTCTGTGTTGGGGCCCCGC	8	14.03	−5.2
2	GATCGATAACTACCCCGAATAACAGGGGACGAGAAT	1	1.75	−7.8
3	TGCAAGTTGGCGGGGCCCCAACA	1	1.75	−4.7
4	TGTTGGGGCCCCGCCAACCTGCA	8	14.03	−5.5
5	TTCTTTATGTTGGGGCCCCGCCAACT	8	14.03	−5.9
6	TGTTGGGGCCCACACCCCAACTTGCA	2	3.51	−12
7	TGCAAGTTGGCGGGGCCCCAACACAGAAGCT	2	3.51	−4.7
8	TGCAAGTTGGCGGGGCTCCAACA	1	1.75	−4.5
9	CAGCTTCTGTGTTGGGGCCCCGCC	1	1.75	−5.2
10	TTCTCTATGTTGGGGCCCCGCCAA	1	1.75	−2.7
11	TCTATGTTGGGGCCCCGCCAACTTG	7	12.28	−5.9
12	TGTTGGGGCCCACACCCCAACTTGCA	1	1.75	−12
13	TGCAAGTTGGCGGGGCCCCAACATAGA	1	1.75	−4.7
14	GATCGATAACTACCCCGAAGAATAGGGGACGAGAAC	1	1.75	−7.8
15	TGTTGGGGCCCCGCCAACCTGCA	2	3.51	−5.5
16	ATTCGATAACTACCCCCGTAGAAGAGGGGACGAGAACT	1	1.75	−8.2
17	CAAGTTGGCGGGGCCCCAACACAGA	1	1.75	−4.7
18	TCTATGTTGGGGCCCCGCCAACTTG	2	3.51	−5.9
19	TGTTGGGCCCCACCCCAACTTGCA	1	1.75	−8.3
20	TGCAAGTTGGCGGGGCCCCAACATAG	1	1.75	−4.7
21	ATGCAAGTTGGGGTGGGGCCCCAACA	2	3.51	−13.2
22	TATTCGATAACTACCCCGAAGAA	1	1.75	−1
23	TGCAAGTTGGCGGGGCCCCAATATAGA	1	1.75	−2.9
24	TGCAAGTTGGCGGGGGCCCAACATAGA	1	1.75	−6.4
25	TATGTTGGGGCCCCGCCAACTTGCA	1	1.75	−5.9

### Spacers

**Figure 2:**
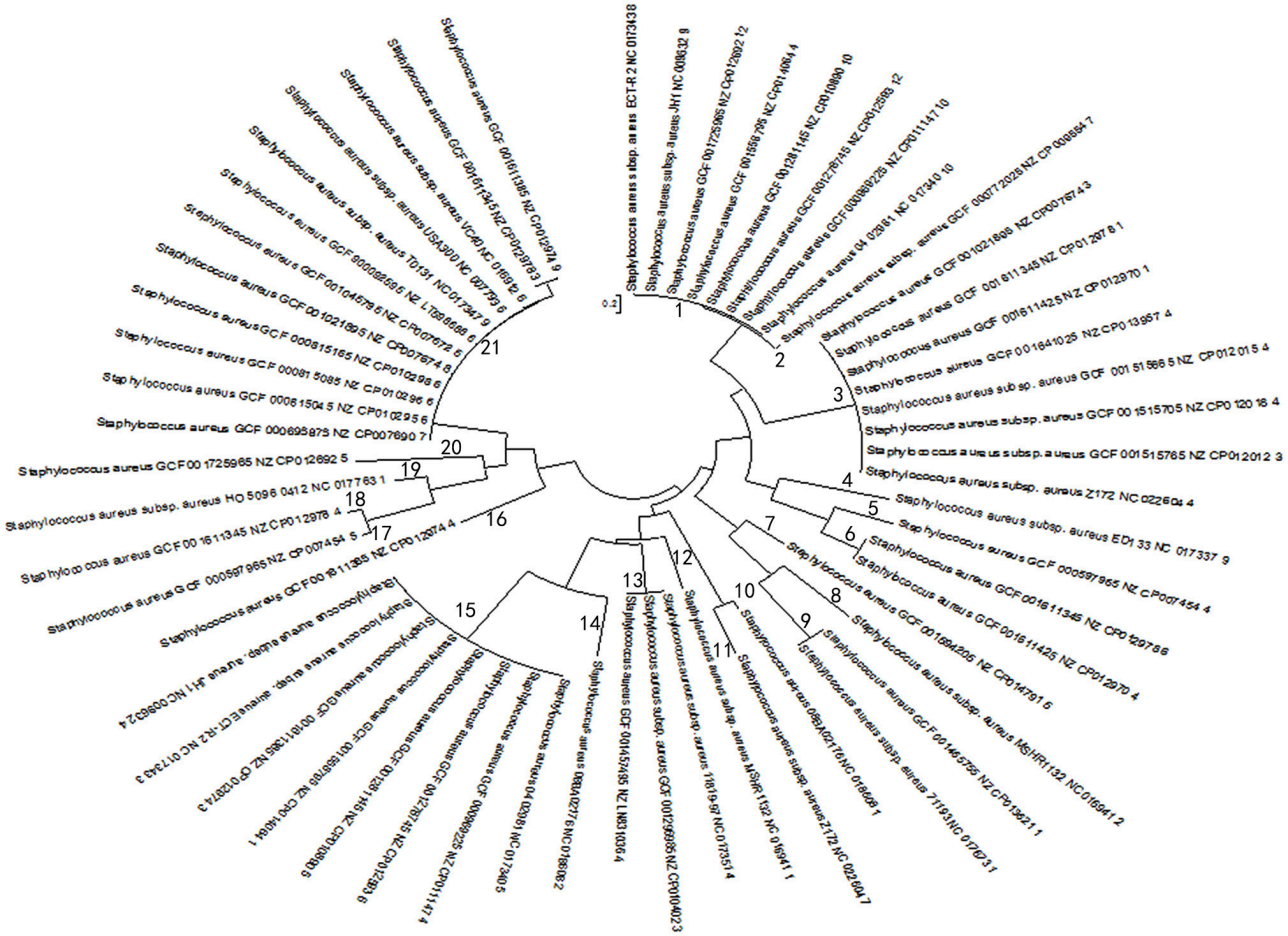
Biological evolution tree generated by principal component spacer sequences. Evolutionary tree results are grouped based on evolutionary relationships. The numbers from 1 to 21 represent 21 groups. Strains in the same group indicate higher evolutionary similarity, and the closer the distance, the higher the affinity is. The evolutionary distance scale is 0.2.

Based on multi-sequence alignment of spacer sequences, a total of 92 spacers were found in 57 CRISPR loci in 38 stains of *S. aureus* and the spacer sequences present at the CRISPR locus appear to be highly homologous, contrary to the diversity necessary as a bacterial self-defense system. But from another perspective, they seemed to have undergone the same phage invasion or the process of gene transfer. After further analysis of all spacers sequences present in the CRISPR system by MSA, the CRISPR loci were classified into 22 groups, What's more, on the basis of the MSA analysis, it was found that there was no highly conserved nucleotide in the spacer sequences of different CRISPR loci. Finally, homologous matching of 92 spacer sequences from 57 CRISPR loci by NCBI blast showed that most of the spacers matched the corresponding exogenous elements. Strikingly, 11 spacers from 2 strains (08BA02176 and MSHR1132) appeared to be highly homologous to exogenous phage or plasmid (Table 3). Besides, PCA was used for each of the 57 CRISPR loci after optimization based on MSA data. In the process of dimensionality reduction of the experimental data, the spacer components of each CRISPR locus were projected onto the same analysis plane for each CRISPR locus, and in the principal component data on the overall contribution of the calculation results were consistent with the required precision. Through such a simplified analysis of the data, it avoided the systematic error of deducting the principle of deduction too much when constructing the phylogenetic tree due to the uneven length of the nucleotide sequence (Moore, 1981). The evolutionary relationship between strains was explored by using the difference between the access and deletion of the spacer sequences. The phylogenetic tree of 57 groups of spacer components was constructed by using mega6.06 software (Figure 2), which enabled the homology analysis of the selected 57 CRISPR loci.

**Table 3:** Statistics of phage or plasmid highly homologous to spacers.

**Stain**	**Genbank ID**	**CRISPR ID**	**SPACER ID**	**Sequence of spacer**	**Similar Phage GI**	**Similar plasmid**
*Staphylococcus aureus* 08BA02176	404477334	NC_018608_1	NC_018608_1-F	TAGAATGTTATTATCTAAGT GGTCGATGTATTCC	735998225, 1352282635, 594138638, 1336442650, 1229407576.	–
			NC_018608_1-G	TCATACTAGCACCCCACTCT CTACTGAACAAGTATCA	765348377	–
			NC_018608_1-H	CTTAAAATCTAATTGCATTG TTATCAATTCCTTTA	1188256656, 558695106, 558694899.	–
			NC_018608_1-K	TTTTCTTTAACTGTTTTTACT GCCCATTTAATAGT	735998439,525336474.	–
			NC_018608_1-M	AAGTTAACGGCATTACCTAA TAAAAATATTTTAGG	584590862, 1345606604, 1332563252, 695256149, 365189246, 365189224.	–
			NC_018608_1-N	TCATCTTTCATGTCACTGAT TAATTCATTTGTA	–	Plasmid SAP020A
			NC_018608_1-O	GGTAATAGTTGCTCAATAGG TAATAAAACGTCGGT	–	Plasmid pAYP1020
		NC_018608_2608_2	NC_018608_2-B	GATATACTCCTTTACCATGT ATTAATTCTGGACCAC	1220001744, 1188256199, 1220003875.	–
*Staphylococcus aureus* subsp. *aureus* MSHR1132	379794527	NC_016941_1	NC_016941_1-D	GTTTTTCATAGTTAATCAAT CCCTTTTCTTTTTT	1192700659,1102331716, 797192878,410809112, 398255565,670139430, 1215500049,1188256881.	–
			NC_016941_1-E	TTAAATCTTTGATTGCTCTT AGCTCTAGTTATGTAT	806933942,1336445532, 1321071118,1321070986, 940328084, 1168037548, 1072301026, 857291865.	–
			NC_016941_1-F	CACGCTGTAGTGAAGTATA GAAACGGCATGAGTACAAT	1321071610,589626950, 402761649, 514343602, 398256436,215260398, 475990627,456174244, 349732033,302749846.	–

### *Cas* genes near CRISPR loci

**Figure 3:**
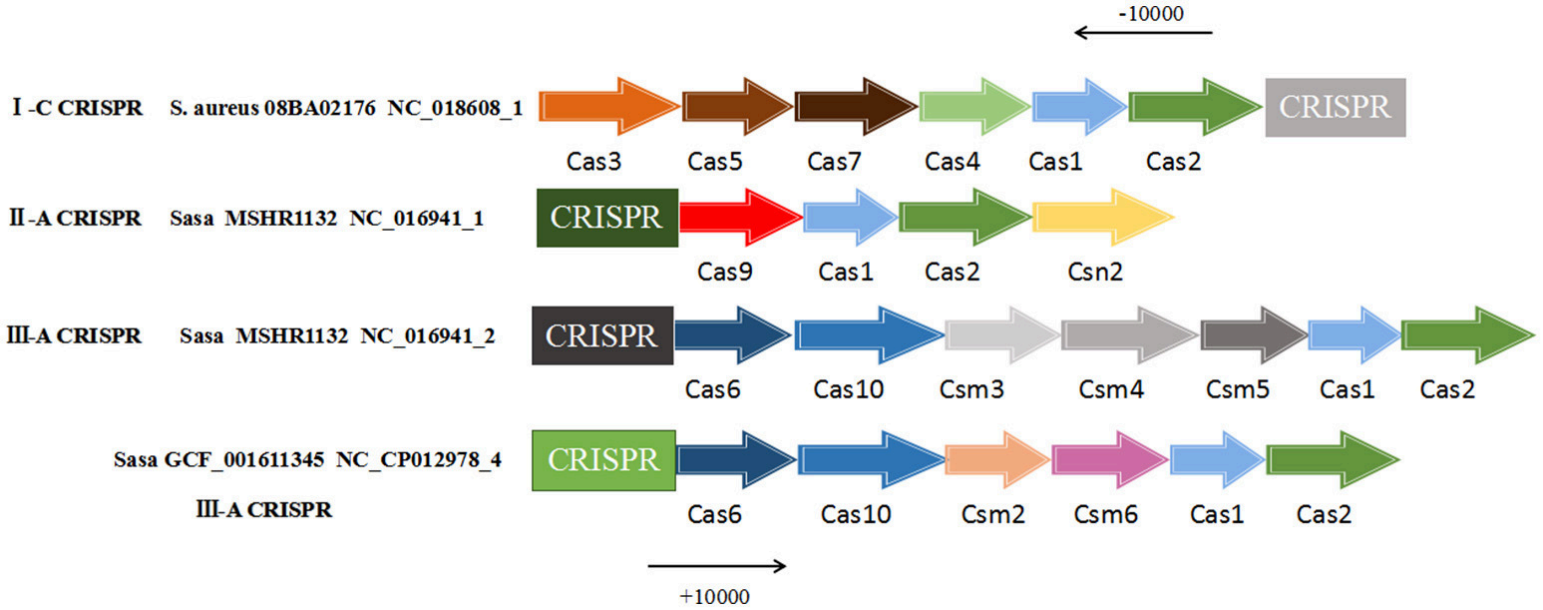
The *cas* genes in vicinity of CRISPR loci. *Cas* genes were searched from 10,000 bp upstream to 10,000 bp downstream the CRISPR sequence. “Sasa” represents *Staphylococcus aureus* subsp. *aureus*. “S. aureus” represents *Staphylococcus aureus*.

For the CRISPR-Cas system, the *cas1* and *cas2* genes were essential elements of a normally active CRISPR system and were located near the CRISPR locus. Therefore, the presence of the *cas1* and *cas2* genes were searched in the range of 10,000 bp upstream and 10,000 bp downstream of all 57 CRISPR loci. The result found that the presence of two core *cas* genes only 3 strains of *S. aureus* were *S. aureus* 08BA02176 (CRISPR ID: NC_018608_1) and *S. aureus* GCF_001611345 (CRISPR ID: NC_CP012978_4) and *S. aureus* MSHR1132 (CRISPR ID: NC_016941_1, NC_016941_2), the other 35 strains did not exist in these two kinds of *cas* gene. Based on this result, *cas* genes contained in these three strains were described (Figure 3). The CRISPR of *S. aureus* 08BA02176 NC_018608_1 belongs to the subtype I-C, its related proteins near CRISPR were endonuclease Cas1, integrase Cas2, helicase Cas3, protein Cas4, protein Cas5 and protein Cas7. The CRISPR of *S. aureus* MSHR1132 NC_016941_1 belongs to the subtype III-A and the CRISPR-related proteins in the vicinity of NC_016941_1 were nuclease Cas9, endonuclease Cas1, integrase Cas2 and protein Csn2. CRISPR's associated proteins near CRISPR of MSHR1132 NC_016941_2 (subtype III-A) were Cas6, protein Cas10, protein Csm10, protein Csm4, endonuclease Cas1 and integrase Cas2. CRISPR-related proteins near CRISPR (GCF_001611345 NC_CP012978_4, subtype III-A) were endonuclease Cas1, integrase Cas2, protein Cas6, protein Cas10, protein Csm2, and protein Csm6. From the observation, it showed that similar proteins of the same CRISPR type can be found on CRISPR loci with 4 or more flanking proteins.

## Discussion

*Staphylococcus aureus* is currently one of the major pathogenic microorganisms that cause infectious diseases in humans worldwide. In recent years, due to the widespread use of multiple antibiotics, various resistance genes (such as mec gene-encoding penicillin-binding protein) were horizontally transferred between *Staphylococcus* strains resulted in multi-drug resistance of *S. aureus* (Chuang et al., 2014; Lin et al., 2016; Miao et al., 2016). One study analyzed 370 CRISPR-Cas systems from 148 prokaryotic genomes and constructed a phylogenetic tree based on the cas1, cas2, cas3, and cas4 genes present in these systems. Surprisingly, the species that are supposed to be closely related to each other are on separate branches of the phylogenetic tree. Further analysis revealed 10 large plasmids with a CRISPR system, suggesting that the CRISPR-Cas system was a mobile genetic element and that many horizontal gene transfer events have occurred through binding transfer, allowing the system to spread among distantly related species (Borowski, 2017). In addition, Bikard et al. (2012) constructed a CRISPR locus targeting a drug resistance gene on the chromosome of the recipient bacteria and introduced the plasmid containing the CRISPR gene into the chromosome of the recipient bacteria, resulting in the death of the bacteria containing the drug resistance gene. Based on this, a mobile CRISPR element targeting a drug-resistant or virulence gene was designed to not only kill pathogens with the target genes but also prevent the spread of drug-resistant or virulence genes among bacteria. The development of this mobile CRISPR element as an antimicrobial agent will provide a new strategy for the control of foodborne pathogens.

Some studies have found that about 40% of the bacterial genome contains the CRISPR locus (Hakim et al., 2016). Most of the CRISPR loci of prokaryotes are located on their chromosomes, rarely on plasmids (Sorek et al., 2008). The main reason for this phenomenon is that the CRISPR system could provide the scavenging effect on the foreign bacteriophage and plasmid through the targeting interference mechanism of RNA, which endowed prokaryotes with strong adaptability in the face of different evolutionary environments (Koonin and Wolf, 2015). If CRISPR exists on the plasmid, it will be detrimental to the heritability of this immunity. In this study, there were 16 kinds of *S. aureus* in all 38 kinds of *S. aureus* contains 2 or more CRISPR loci, and they may present different CRISPR system type. It directly proved that a bacterial genome containing multiple CRISPR loci. There were great distinctions in the number and characteristics of the CRISPR loci contained among different species, which was also a necessary condition for bacterial genotyping (Horvath et al., 2009). In general, direct repeats of a single CRISPR locus are extremely conserved; however, there exist in some modified nucleotides in direct repeats within different CRISPR loci. When a new spacer is formed, it always remove the internal spacer by homologous recombination between direct repeats to limit the size of the CRISPR. The terminal repeats were frequently observed to be polymorphic, which was well-explained by the frequent loss of spacer/repeats containing the last of the terminal repeats (Vahidi and Honda, 1991; Horvath et al., 2008). The position of the repeat appears to be consistent with the location of nearby cas gene. In addition, variations within repetitive sequences often occur throughout the CRISPR locus (Horvath et al., 2008). In the 38 strains of *S. aureus*, repeats of most strains were highly conserved and were located in groups 1, 4, and 5. Therefore, it could be inferred that these direct repetitive mutations were less likely to occur in other groups, suggesting that the presence of cas around the CRISPR locus in these clusters is probably less.

Due to the presence of short palindromic sequences in direct repeats, the secondary structures of RNA that may be formed by these direct repeats during transcription were investigated. This structure could cooperate with the crRNA transcribed from the entire CRISPR sequence to form a bimodal structure, guiding the Cas protein to target the site. Kunin et al. (2007) indicated that the stem-loop structure of some repeats may contribute to recognition-mediated contact between a gap-targeted exogenous RNA or DNA and a Cas-encoded protein, suggested that the stability of RNA secondary structure may affect CRISPR function. In addition, the MFE values of RNA secondary structures formed by all 25 different direct repeats were predicted. By comparing their MFE, secondary structures of RNA with lower minimum free energies were more stable. The stability of secondary structure of RNA was found depends on the length of the repeat and by the number of bp as well as the content of “GC” in stem. By homologous comparison of the spacer sequences in the NCBI Database, the source of the known genes highly homologous thereto was queried. Boch et al. (2009) indicated that the spacer sequence was proved from the exogenous gene components, CRISPR got the spacer sequence from the new invasion of exogenous DNA by some means. A homology survey of all the spacer sequences of this experiment by NCBI blast showed that most of the spacers of *S. aureus* strains can be matched to the foreign phage or plasmid in NCBI. In particular, 11 spacers from 2 strains of *S. aureus* presented highly matched exogenous phage or plasmids (as shown in Table 3). This strongly proves that the spacer is derived from exogenous elements. To a certain extent, the higher the number of phages with higher similarity to the spacers, the higher the frequency of phages attacking to the bacterium, and the more important the survival of the CRISPR system for bacteria in the environment of phage invasion. In addition, the phylogenetic tree of the selected strains was constructed by using the spacer sequences to provide reference value for the genotyping of *S. aureus*. In fact, the CRISPR-based typing has been well-established in some bacteria, the most representative of which is the application of *Salmonella* typing (Liu et al., 2011; Fabre et al., 2012). Even recent studies have demonstrated that Helicobacter pylori can be typed using CRISPR-like sequences in combination with virulence genes. The CRISPR-virulence technique was constructed for the 20 Helicobacter pylori strains obtained and compared with the phenotype obtained by the random amplified polymorphic DNA technique. There is no difference between the discrimination of the CRISPR typhoid typing and the RAPD typing (Bangpanwimon et al., 2017). Thus, the CRISPR-based typing method has a broad application prospect in the investigation of foodborne pathogens. However, at the moment, the data of the research on the typing of foodborne pathogenic bacteria by this method is still not enough, the database is not perfect, and no uniform standard. Due to the increasingly serious *S. aureus* infection and the relatively few studies on the CRISPR of *S. aureus*, which means that extensive research on the CRISPR typing of *S. aureus* is particularly urgent. Through the establishment of a database to explore the occurrence of foodborne pathogens CRISPR, diversity and activity in the relevant pathogens, in order to solve the problem of lasting food safety (Nikki and Dudley, 2014).

In this paper, the 92 spacers were found in 38 species of *S. aureus*, of which only 9 species of *S. aureus* contained 2 or more spacers, and other strains containing only one spacer, the 92 spacers with an average length of about 32 bp, the shortest 25 bp, the longest 51 bp, and the diversity of spacer length and sequence will affect the bacterial activity in the CRISPR system. Horvath et al. (2008) studies showed that longer CRISPR sequences may be more active than short ones. Di et al. (2014) studies indicated that CRISPR loci containing more number of the spacers with length of 30 bp were more active than sites containing a small amount of 36 bp. Therefore, the CRISPR loci in the strains of our choice may be more active than the previously studied strains with shorter spacers. Additionally, the same nucleic acid sequence was observed in the spacers of different strains, which suggested that these strains may be attacked by several phages with higher relatives or due to horizontal gene transfer.

For the 57 CRISPR loci contained in 38 *S. aureus* strains, each of the 10,000 bp upstream and downstream of these CRISPR loci was queried for the presence of *cas1* and *cas2* genes. Three *S. aureus* strains were screened to meet the requirements. The three *S. aureus* strains contained *cas1* and *cas2* genes to systematically search for the distribution of other *cas* genes in the vicinity of the CRISPR sequence. Wherein in the CRISPR system of *S. aureus* 08BA02176, the *cas* gene have located in 10,000 bp downstream of the CRISPR sequence. However, in the two other strains of *S. aureus* (*S. aureus* GCF_001611345 and *S. aureus* subsp. *aureus* MSHR1132), the *cas* gene have located in upstream of the CRISPR sequence, it proved that CRISPR-Cas system could transfer between the same strains (Borowski, 2017). In summary, the other strains of *S. aureus* that did not detect the *cas1* and *cas2* genes were considered inactive by the CRISPR system they contained, because when these key *cas* genes in certain CRISPR loci inactivated or not present, bacterial drug resistance and its ability to integrate new spacer sequence will be lost.

At present, research on the CRISPR system of pathogenic bacteria of foodborne origin has not paid enough attention. In fact, in addition to the immune defense function, the CRISPR system has also been found to have the functions of regulating the virulence of bacteria and influencing the formation of biofilms of foodborne pathogens. Zegans et al. (2009) found that the CRISPR system was involved in the lysogenic infection of bacteriophage DMS3 in *Pseudomonas aeruginosa PA14*, resulting in the inhibition of *P. aeruginosa* biofilm formation and decreased the swarming motility. Palmer and Gilmore (2010) indicated that multidrug-resistant *Enterococcus* strains were usually missing the CRISPR system, and thus speculated that the CRISPR system may be involved in the interference of drug-resistance gene capture. Lovley and Muktak (2010) showed that the spacer1 in the CRISPR system is homologous to the histidine-tRNA synthetase of the bacterium in *Pelobacter carbinolicus*, which prompted *P. carbinolicus* to selectively delete the genes with more histidine codons during evolution. As a result, *P. carbinolicus* differentiated from other geobacteraceae to form new species. Babu et al. (2011) demonstrated that Cas1 (Ygb T) in the CRISPR I-E system of *E. coli* K12 can act on the ss DNA or branched DNA in the Holiday model, replication fork, 5′-flaps structure, and cleave them to affect the recombination and repair of the host genome. These studies revealed that the CRISPR system still has many unknown functions in regulating bacterial physiological activities. Most of the current research is based on bacterial genotyping, drug resistance, epidemiological studies, and traceability analysis. Therefore, in this study, *S. aureus*, the representative of foodborne pathogens, was selected as the research target, and the basic structure of the CRISPR system contained in the 38 strains of *S. aureus* was analyzed by means of bioinformatics. The results of bioinformatics analysis can provide a data reference for a broader range of CRISPR studies in *S. aureus*. At present, there are still many foodborne pathogens CRISPR system research is not involved, which need to be based on large data genotyping and foodborne pathogenic mechanism of pathogenesis is unfavorable, in this regard, put more effort to construct extensive research on CRISPR system of food borne pathogenic bacteria is imperative. With the rapid development of CRISPRs technology, from the CRISPR-Cas9 DNA-editing system to the discovery of the CRISPR-Cas13 RNA-editing system that is now replacing RNAi technology (Doudna and Charpentier, 2014; Abudayyeh et al., 2017), there is no doubt that CRISPR system have underestimated in the future various fields of research potential and application value. It is believed that as the structure of the CRISPR system is further revealed, which will change the research pattern in the life sciences.

## Conclusion

Most strains of *S. aureus* contain only one CRISPR site, and a few strains contain multiple sites with sparse distribution. These loci mainly include highly conserved direct repeats and highly variable spacers, as well as polymorphic *cas* genes. In addition, all direct repeats can form stable RNA secondary structures, and spacer sequences have been shown to originate from exogenous phage or plasmids. Moreover, the specificity of spacer sequences can serve as a basis for accurate genotyping techniques. Three different CRISPR system subtypes were found in 38 *S. aureus* strains, including 4 active CRISPR-Cas systems. The analysis and comparison of the CRISPR/Cas system can help to understand the environmental adaptability of each *S. aureus* evolutionary lineage that represents different pathogenicity. Bioinformatics analysis provides data support for bacterial typing, traceability analysis, and exploration of CRISPR other than immune. The CRISPR study provides new ideas for preventing the spread of resistant genes between *S. aureus* and eliminating drug resistance genes.

## Author contributions

XZ designed this study and wrote the manuscripts. ZY finished the experiments and collected the data. XZ, ZY, and ZX revised the manuscript critically for important intellectual content. All authors read and approved the final manuscript.

### Conflict of interest statement

The authors declare that the research was conducted in the absence of any commercial or financial relationships that could be construed as a potential conflict of interest.
